# Effects of dexmedetomidine and propofol on patient-ventilator interaction in difficult-to-wean, mechanically ventilated patients: a prospective, open-label, randomised, multicentre study

**DOI:** 10.1186/s13054-016-1386-2

**Published:** 2016-07-02

**Authors:** Giorgio Conti, Vito Marco Ranieri, Roberta Costa, Chris Garratt, Andrew Wighton, Giorgia Spinazzola, Rosario Urbino, Luciana Mascia, Giuliano Ferrone, Pasi Pohjanjousi, Gabriela Ferreyra, Massimo Antonelli

**Affiliations:** Department of Intensive Care and Anaesthesia, Catholic University of Rome, Policlinico A. Gemelli, Largo F. Vito 1, Rome, 00168 Italy; Department of Anaesthesia and Intensive Care Medicine, Sapienza University of Rome, Policlinico Umberto I, Viale del Policlinico 155, Rome, Italy; Orion Pharma R&D, Nottingham, UK; Department of Anaesthesia and Intensive Care Medicine, S. Giovanni Battista Molinette Hospital, University of Turin, Corso Dogliotti 14, Turin, 10126 Italy; Orion Pharma R&D, Kuopio, Finland

**Keywords:** Sedation, Mechanical ventilation, Propofol, Dexmedetomidine, Patient-ventilator synchrony

## Abstract

**Background:**

Dexmedetomidine can be used for sedation of mechanically ventilated patients and has minor respiratory effects. The aim of this study was to compare the incidence of patient-ventilator dyssynchronies during sedation with dexmedetomidine or propofol.

**Methods:**

We conducted a multicentre, prospective, open-label, randomised clinical trial, comparing dexmedetomidine with standard propofol sedation at three intensive care units of university hospitals in Italy. Twenty difficult-to-wean patients for whom the first weaning trial had failed and who were on pressure support ventilation were randomised to receive sedation with either dexmedetomidine or propofol at a similar level of sedation (Richmond Agitation-Sedation Scale [RASS] score +1 to −2). The asynchrony index (AI) was calculated using tracings of airflow, airway pressure and electrical activity of the diaphragm sampled at 0, 0.5, 1, 2, 6, 12, 18 and 24 h.

**Results:**

The mean AI was lower with dexmedetomidine than with propofol from 2 h onwards, although the two groups significantly differed only at 12 h (2.68 % vs 9.10 %, *p* < 0.05). No further difference was observed at 18 and 24 h.

**Conclusions:**

When sedation with propofol and dexmedetomidine was compared at similar RASS scores of patients in whom first weaning trial had failed, the AI was lower with dexmedetomidine than with propofol, and this difference was statistically significant at 12 h. These results suggest that sedation with dexmedetomidine may offer some advantages in terms of patient-ventilator synchrony.

## Background

Patients receiving assisted mechanical ventilation (MV) commonly require sedation to optimize tolerance to the endotracheal tube and to better adapt to the ventilator, thus decreasing stress response, anxiety and discomfort [1–5]. Use of sedation to optimize the patient-ventilator interaction can help avoid prolongation of MV and intensive care unit (ICU) length of stay as well as an increased need for tracheostomy [6, 7].

For several decades, γ-aminobutyric acid receptor agonists (including propofol and benzodiazepines) have been the most commonly used sedatives for critically ill patients, including those receiving assisted MV [1, 4, 5, 8]. During assisted MV, patient-ventilator interaction is influenced both by machine settings [6, 7] and by the patient’s respiratory pattern, timing and drive [8, 9]. These are directly affected by sedatives, whose effects vary, depending both on the drug used and on the dose administered [10, 11]. Very recently, research has shown that sedation with propofol was associated with a reduction in respiratory drive causing significant derangements in patient-ventilator synchrony in ICU patients receiving assisted ventilatory support [9]. As part of that research, the electrical activity of the diaphragm (EAdi) signal, determined using a nasogastric feeding tube with a multiple array of electrodes placed at its distal end, was successfully used to assess respiratory drive, neural respiratory rate and neural timing [9]. That technique enabled an easy computation of the asynchrony index (AI), a metric previously reported to be an independent predictor of longer MV and ICU stay [6].

Because dexmedetomidine is an α_2_-adrenoceptor agonist that provides sedation and anxiolysis via receptors in the locus coeruleus [10], as well as analgesia via receptors at the spinal cord level, it is able to attenuate the stress response without significant respiratory depression [11–15]. This pharmacological profile may be of particular interest in patients receiving assisted MV because it may possibly reduce the rate of patient-ventilator asynchronies directly generated by the influence of sedatives on the output of the respiratory centres, which can affect the respiratory drive and/or timing.

In two double-blind, prospective, randomised clinical trials, researchers recently compared dexmedetomidine with propofol and midazolam in mechanically ventilated critically ill patients. Both studies showed that dexmedetomidine is an effective sedative agent compared with propofol and midazolam, and that its use is associated with easier communication with patients, better assessment of pain, reduced delirium and decreased time to extubation compared with propofol [16].

We hypothesized that dexmedetomidine may reduce the incidence of patient-ventilator dyssynchronies compared with propofol. To examine this hypothesis, we conducted a multicentre, prospective, open-label, randomised clinical trial, comparing dexmedetomidine against standard sedation with propofol infusion.

## Methods

### Patients

Adult ICU patients who had failed one weaning trial were recruited. For the purposes of this study, difficulty to wean was determined by failure at a single adequate weaning trial, according to a common weaning protocol based on the progressive reduction of the pressure support (PS) level and ending with a spontaneous breathing trial of 30 minutes at 7 cmH_2_O of pressure support ventilation (PSV) and positive end-expiratory pressure (PEEP) of 5 cmH_2_O, using the following standard criteria [17]:

1. Respiratory rate >35 breaths/minute
2. Partial pressure of oxygen (PaO_2_) <65 mmHg with fraction of inspired oxygen (FiO_2_) <0.5 or oxygen saturation (SaO_2_) <90 %
3. Partial pressure of carbon dioxide (PaCO_2_) >50 mmHg or an increase in PaCO_2_ > 8 mmHg
4. pH <7.32 or decrease of ≥0.07 pH units
5. Evident respiratory distress (diaphoresis, accessory muscle recruitment, thoracoabdominal paradox)
6. Heart rate (HR) >140 beats/minute or a sustained increase or decrease in HR >20 %
7. Severe arrhythmias
8. Systolic arterial pressure <90 mmHg or >180 mmHg
9. Coma, agitation or anxiety.

Patients who had been intubated and mechanically ventilated in the ICU for >24 h and who had received propofol as the sole agent for continuous sedation (minimum 12 h) with a target sedation level of +1 to −2 on the Richmond Agitation-Sedation Scale (RASS) were included in the study.

Patients who had already failed more than one weaning trial were excluded. Other key exclusion criteria were as follows:

1. Acute severe intracranial or spinal neurological disorder
2. Uncompensated acute circulatory failure
3. Severe bradycardia

### Study design and procedures

We conducted a phase IIIb, multicentre, prospective, randomised, open-label, comparator-controlled study. The ethics committees of the University of Turin and the Catholic University of Rome approved the protocol, and informed consent was obtained from all patients. The protocol was recorded in the European Clinical Trials Database (EudraCT 2011-001490-40).

### Equipment

MV was applied using a Servo-i ventilator equipped with NAVA software (Maquet Critical Care, Solna, Sweden). EAdi was determined using a nasogastric feeding tube with a multiple array of electrodes placed at its distal end (EAdi catheter; Maquet Critical Care). Correct positioning of the EAdi catheter was ensured using a dedicated function of the ventilator, as previously described [18]. During the study, all patients were mechanically ventilated in PSV mode (range 10–18 cmH_2_O) with a PEEP of 5–8 cmH_2_O and an FiO_2_ ranging between 0.35 and 0.5. Attending physicians optimized the ventilator settings according to a protocol based on clinical response, blood gas values and ventilator tracings. The level of PS was titrated to obtain a tidal volume of 6–8 ml/kg with active inspiration [9]. Servo-i default trigger settings were used during the study period (inspiratory level 5, corresponding to 50 % of the 2 L/minute bias flow; expiratory 30 % of peak inspiratory flow); in cases of evident short cycling, the cycling off was optimized according to the patient’s EAdi signal. FiO_2_ and PEEP were maintained at the values in use prior to patient enrolment. Ventilator settings were kept as constant as possible during the study.

### Study protocol

Prior to randomisation, each centre enrolled three run-in patients who were treated with dexmedetomidine so that study staff could become familiar with the agent. Neural and respiratory parameters were not collected from these patients, who were included only in the safety analyses.

For the first 24 h after the start of the study treatment, periodic measurements of EAdi, respiratory parameters and arterial blood gases were assessed to compare the effects of dexmedetomidine and propofol on ventilation. During this period, 10-minute samples were taken at 0, 0.5, 1, 2, 6, 12, 18 and 24 h, and all breaths were analysed for evidence of asynchrony. For each patient, we also measured the average peak EAdi (i.e., the maximum level of EAdi generated by the diaphragm) and time of synchrony (i.e., the time in which the mechanical inspiration generated by the ventilator and diaphragmatic contraction were in phase) over 10 minutes of data recording. For determination of the primary endpoint, all of these 10-minute samples were added together to create the AI. The AI is expressed as the percentage of total breaths during the period of interest in which asynchrony is identified. It has previously been analysed in other trials [6, 7] and is expressed as follows:

$$ \mathrm{AI} = \left(\mathrm{number}\ \mathrm{of}\ \mathrm{asynchrony}\ \mathrm{events}/\mathrm{total}\ \mathrm{respiratory}\ \mathrm{rate}\right) \times 100, $$

where the total respiratory rate is obtained from the EAdi tracings and the following asynchronies are considered in the computation of the AI: wasted effort, double-triggering and auto-triggering.

At the end of the 24-h treatment period, a weaning attempt was made. Further attempts were made at least every 24 h until the patient was successfully extubated.

Secondary endpoints included maximum level of EAdi per breath expressed in microvolts (peak EAdi), time of synchrony, respiratory rate, arterial blood gas parameters, time to extubation, duration of MV and duration of ICU stay.

The assigned study treatment was continued until successful extubation, but for no longer than 14 days. Following withdrawal of sedation, patients were monitored for 48 h and either contacted by telephone or assessed at a visit 30 (±5) days after randomisation.

### Drugs

After the index weaning failure, all patients required a continuous infusion of sedatives due to anxiety generated by respiratory distress and endotracheal tube intolerance. Patients were randomised to receive either dexmedetomidine (Orion Pharma, Espoo, Finland), or propofol (purchased within the European Union) intravenously at rates of 0.2–1.4 μg/kg/h and 0.3–4 mg/kg/h, respectively, to maintain the RASS score within the range of +1 to −2. The identity of study sedatives was not blinded to caregivers.

During the course of the study, and especially during the first 24 h, the investigators attempted to manage the sedation of patients using the study drug only. Rescue midazolam was used if needed; however, because it had the potential to interfere with the study measurements, participants were advised that it should be used as sparingly as possible. Non-opioid drugs such as paracetamol were preferred for controlling pain.

### Statistical analysis

The primary efficacy variable, AI, was analysed using a repeated-measures analysis of covariance (ANCOVA) model with the study treatment group, centre and baseline AI as between factors and time as the within factor. Individual time points were compared between the study treatment groups.

The secondary efficacy variables, which comprised other neural and respiratory parameters, were analysed similarly to the AI. For arterial blood gases, the mean change from baseline between treatment groups was compared using repeated-measures ANCOVA, where time was considered as the within factor, treatment as the between factor and baseline as the covariate. Time to extubation and duration of MV were compared between treatment groups by applying the Kaplan-Meier method and Cox proportional hazards regression, respectively.

All randomised subjects who received the study treatment were used to evaluate the efficacy variables. Safety data were evaluated using descriptive statistics for all subjects who received the study medication.

The planned sample size of ten in each group had 80 % power to detect a difference in means of 8.3 %, assuming that the common standard deviation is 6.0, using a two-group *t* test with a two-sided significance level of 0.05. In response to evidence of a non-normal distribution, AI data were transformed before the analysis using a two-parameter version of the Box-Cox transformation.

## Results

In total, 26 patients were enrolled, the first 6 of whom were selected for run-in treatment. The other 20 patients (11 men, 9 women) were randomised to receive either dexmedetomidine or propofol. The patients’ mean (standard deviation [SD]) age was 68.8 (15.7) years (range 39–88 years), and their mean (SD) weight was 77.2 (20.8) kg. All patients were white. The main reason for admission to the ICU was medical (50 %), followed by surgical (35 %) and trauma (15 %). The main medical reason for admission to the ICU was cardiac disorders (ten patients). These predominantly included arrhythmia (five patients in each group), chronic cardiac failure (three in the dexmedetomidine group and one in the propofol group) and myocardial ischaemia and infarction (two in each group).

The median durations of study treatment were 31.5 h (range 18–174 h) in the dexmedetomidine group and 47.9 h (range 22–113 h) in the propofol group. The average doses were 0.46 μg/kg/h in the dexmedetomidine group and 1.08 mg/kg/h in the propofol group. At 30-day follow-up, three patients in the propofol group and four patients in the dexmedetomidine group had died, and one patient in the propofol group was lost to follow-up.

### Efficacy

#### Primary endpoint

**Fig. 1:**
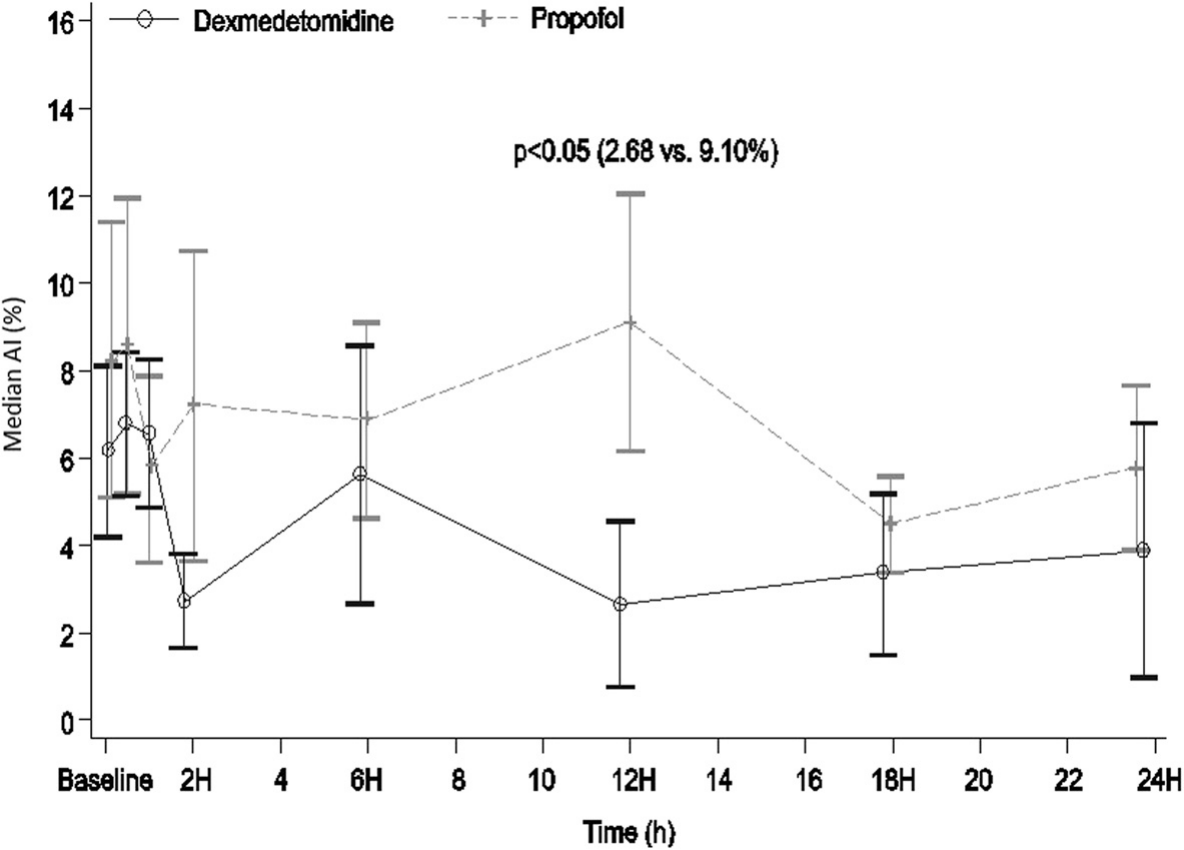
Variation in asynchrony index (AI) with dexmedetomidine and propofol

The mean AI was lower with dexmedetomidine than with propofol from 2 h onwards, although the two groups differed significantly only at 12 h (2.68 % vs 9.10 %, *p* < 0.05) (Fig. 1). No further statistical difference was observed at 18 and 24 h. Of note, in basal conditions, two patients per group had an AI >10 %; at 6 h, two patients in the propofol group and one in the dexmedetomidine group had an AI >10 %; and at 12 and 24 h, four patients in the propofol group and one in the dexmedetomidine group had an AI >10 %.

#### Secondary endpoints

**Fig. 2:**
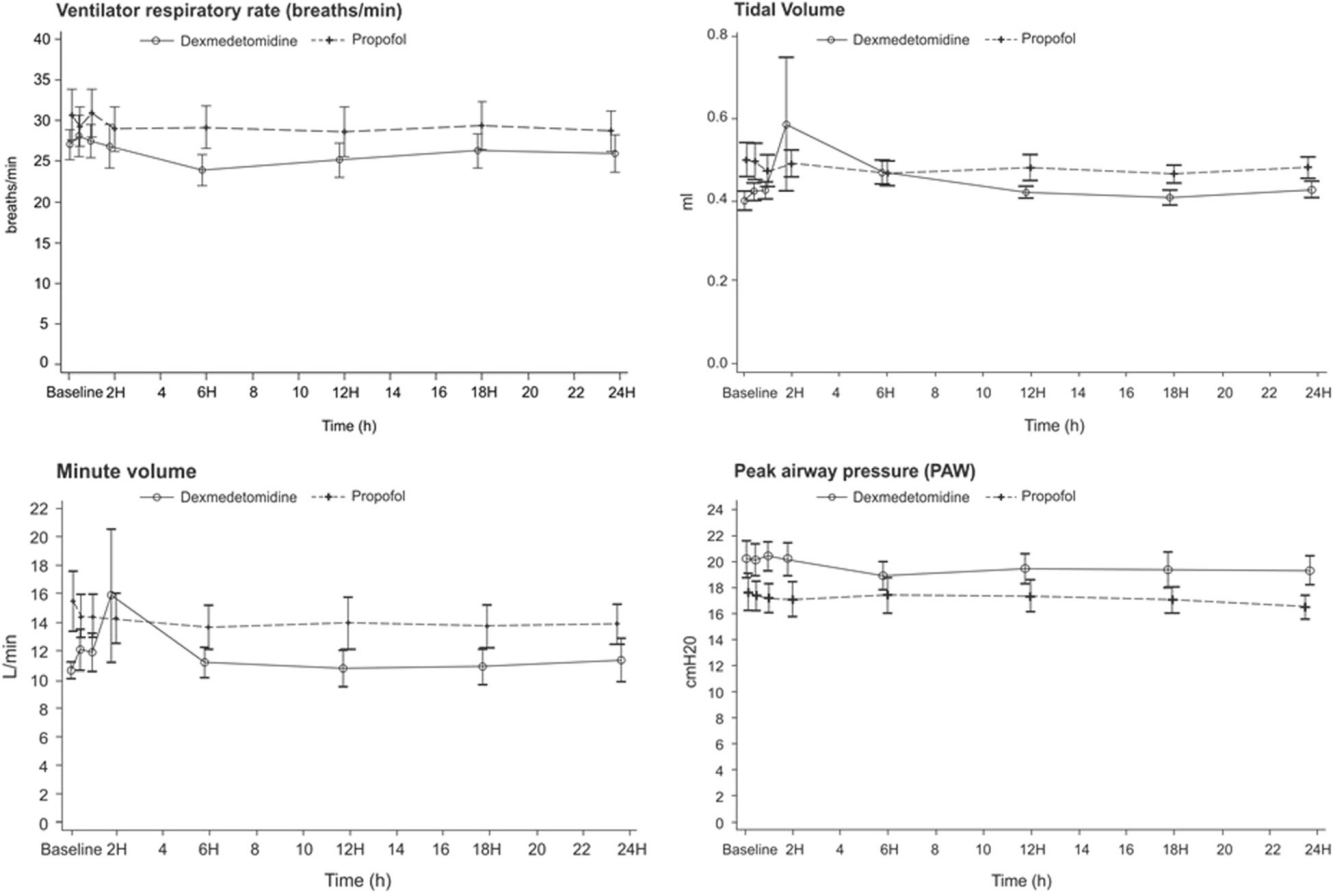
Variation in respiratory parameters with dexmedetomidine and propofol

At similar levels of PSV and PEEP in both groups (PSV 12.4 ± 2.5 cmH_2_O vs 12.9 ± 2.4 cmH_2_O, respectively; *p* = 0.33; PEEP 5.4 ± 1 cmH_2_O vs 5.8 ± 0.9 cmH_2_O, respectively; *p* = 0.17), there were no significant inter-group differences during the first24 h in peak EAdi over time, time of synchrony, tidal volume, minute volume, peak airway pressure, ventilator respiratory rate, mean lactate, PaCO_2_, PaO_2_, SaO_2_ and pH (Fig. 2).

**Fig. 3:**
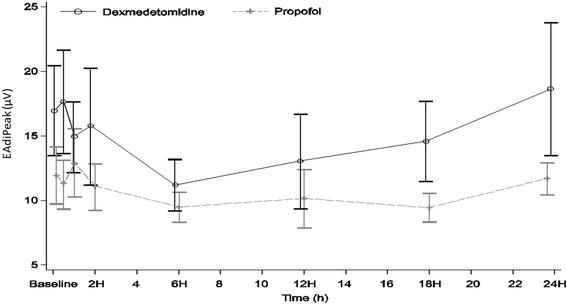
Variation in peak electrical activity of the diaphragm (EAdi) with dexmedetomidine and propofol

There was some imbalance between the two groups regarding peak EAdi at baseline, which persisted at the end of 24 h, but there were no significant differences between the treatments (Fig. 3).

### Time to extubation

The median times to extubation were 25.18 h (range 24.5–118.7 h) in the dexmedetomidine group and 57.33 h (range 24.7–113.0 h) in the propofol group (hazard ratio [HR] 0.974, 95 % confidence interval [CI] 0.373–2.542, *p* = 0.958).

### Duration of ICU stay

The median durations of ICU stay were 6.02 days (range 2.2–8.5 days) in the dexmedetomidine group and 10.06 days (range 5.0–24.8 days) in the propofol group (HR 0.843, 95 % CI 0.305–2.330, *p* = 0.742). Consequently, the average cost of ICU stay was less in the dexmedetomidine group than in the propofol group (€20,387 vs €29,010).

### Episodes of oversedation

All patients remained within the target sedation range (RASS score +1 to −2) during the entire study period, with the exception of two subjects receiving propofol, for whom the RASS score was less than −2 on at least one occasion. There were no subjects with a RASS score less than −2 in the dexmedetomidine group. Midazolam (single 1-mg intravenous bolus) as a sedative was given to two patients in the dexmedetomidine group and to one in the propofol group. For pain control, a fentanyl bolus dose of 50 μg was used in two patients in the dexmedetomidine group and in three in the propofol group.

### Daily weaning trial outcome and reasons for failure

On day 2, nine patients were successfully extubated (six in the dexmedetomidine group and three in the propofol group). Common causes of failure of the weaning trial in both groups are summarized in Table 1. No patient required reintubation in the first 24 h after extubation.

**Table 1:** Common causes of failure of weaning trial on day 2

Cause of failure	Number of patients	
	Dexmedetomidine	Propofol
Respiratory rate >35 breaths/minute	2	4
PaO_2_ <65 mmHg with FiO_2_ <0.5 or SaO_2_ <90 %	1	4
Evident respiratory distress	2	3
Coma, agitation or anxiety	2	3

*FiO*_*2*_ fraction of inspired oxygen, *PaO*_*2*_ partial pressure of oxygen, *SaO*_*2*_ oxygen saturation

### Adverse events

The only treatment-related adverse event observed during the study in the dexmedetomidine group was bradycardia in one patient. In the propofol group, one individual prematurely discontinued the study due to decreased cough efficiency and increased bronchial secretion, and one died as a result of sudden cardiac arrest (Table 2).

**Table 2:** Summary of adverse events observed during the study

Adverse event	Dexmedetomidine (*n* = 16)		Propofol (*n* = 10)		Total (*n* = 26)	
	Subjects, *n* (%)	Events, *n*	Subjects, *n* (%)	Events, *n*	Subjects, *n* (%)	Events, *n*
Bradycardia	1 (6.3)	1			1 (3.8)	1
Cardiac arrest			1 (10.0)	1	1 (3.8)	1
Urinary tract infection	1 (6.3)	1			1 (3.8)	1
Decreased cough efficiency			1 (10.0)	1	1 (3.8)	1
Increased bronchial secretion			1 (10.0)	1	1 (3.8)	1

## Discussion

This study produced indications that, after optimization of ventilator settings according to clinical response, blood gases and ventilator tracings for the same level of sedation, the AI was lower with dexmedetomidine than with propofol. The difference was small; however, it was statistically significant only at the 12-h time point and emerged from a small patient sample characterized by a high degree of variation and a non-normal distribution. As such, our findings regarding AI should be regarded as suggestive rather than conclusive. Additional clinical studies with larger populations and/or datasets are needed to develop our observations.

Sedatives influence the output of the respiratory centres by affecting the respiratory drive and/or timing. Assessment of EAdi allowed Vaschetto et al. [9] to show that, at doses determining deep sedation, propofol reduced neural drive and effort while not significantly affecting respiratory timing and therefore produced a significant deterioration in patient-ventilator synchrony.

Dexmedetomidine, an α_2_-adrenoceptor agonist, does not induce respiratory depression and has not been associated with any effect on patient respiratory activity [12–15]. Moreover, recent studies [16, 19–21] have demonstrated that dexmedetomidine, by virtue of its pharmacodynamic properties, appears to shorten the duration of MV compared with midazolam and the time to extubation compared with both midazolam and propofol.

Optimized interaction with the mechanical ventilator is an important aspect of assisted ventilatory support. Poor patient-ventilator interaction causes discomfort and dyspnoea [22–26], increases the need for sedative and paralytic agents [27], prolongs MV duration and ICU length of stay [6, 28, 29], and increases both the likelihood of respiratory muscle injury [30, 31] and the need for tracheostomy [6].

Several groups of authors have directly analysed the clinical effects of a high rate of asynchronies during assisted MV. Thille et al. [6] showed that ineffective expiratory efforts and double-triggering accounted for >98 % of all asynchronies and that an AI >10 % was associated with a longer duration of MV, whereas de Wit et al. [28] found that an ineffective triggering index >10 % was an independent predictor of longer MV duration and ICU stay.

Very recently, Blanch et al. [32], in a study in which 50 patients were evaluated continuously for >7027 h using specific automatic software for detecting asynchronies, partly confirmed these data, showing that an AI >10 % was associated with a trend towards a longer duration of MV and significantly higher ICU and hospital mortality. In more detail, ICU mortality was 14 % vs 67 % (*p* < 0.01) and hospital mortality was 23 % vs 67 % (*p* = 0.04) in a direct comparison of patients with an AI < 10 % with patients with an AI >10 %.

Strengths of the present study include the use of objective variables to assess the primary endpoint (AI) and that the two regimens were compared at similar sedation levels and at similar levels of ventilator assistance and PEEP. Some limitations must be taken into account, however. First, only 20 patients were enrolled, which represents too small a sample to establish statistically robust and conclusive findings. Second, staff treating the patients were not blinded regarding the sedative administered. However, to limit the influence of this drawback, the researchers who analysed the patients’ respiration tracings were blinded regarding the choice of sedative.

## Conclusions

Our data indicate that when sedation with propofol or dexmedetomidine was directly compared at similar levels of sedation (RASS score −1 to +2), and at similar levels of PSV and PEEP in a group of patients for whom the first weaning trial had failed and were therefore at risk of an increased rate of patient-ventilator asynchrony, there were no significant between-group differences in terms of peak EAdi, minute volume, ventilator respiratory rate, arterial blood gases, rate of adverse events, length of stay in the ICU or time of extubation. Conversely, the AI was lower with dexmedetomidine than with propofol from 2 h onwards, although the two groups differed significantly only at 12 h. The results of our study suggest that, after optimization of ventilator settings, sedation with dexmedetomidine could offer some advantages in terms of patient-ventilator synchrony, but additional clinical studies with larger populations are needed to confirm this postulation.

## Key messages

- When sedation with propofol or dexmedetomidine was compared at similar Richmond Agitation-Sedation Scale scores in patients for whom the first weaning trial had failed, the asynchrony index was lower with dexmedetomidine, and this difference was statistically significant at 12 h.
- These results suggest that sedation with dexmedetomidine could offer some advantages in terms of patient-ventilator synchrony.

## References

[1] Sydow M, Neumann P (1999). Sedation for the critically ill. Intensive Care Med..

[2] Brook AD, Ahrens TS, Schaiff R, Prentice D, Sherman G, Shannon W (1999). Effect of a nursing implemented sedation protocol on the duration of mechanical ventilation. Crit Care Med..

[3] Kress JP, Pohlman AS, O’Connor MF, Hall JB (2000). Daily interruption of sedative infusions in critically ill patients undergoing mechanical ventilation. N Engl J Med..

[4] Swart EL, Zuideveld KP, de Jongh J, Danhof M, Thijs LG, Strack van Schijndel RM (2006). Population pharmacodynamic modelling of lorazepam- and midazolam-induced sedation upon long-term continuous infusion in critically ill patients. Eur J Clin Pharmacol.

[5] Barr J, Egan TD, Sandoval NF, Zomorodi K, Cohane C, Gambus PL (2001). Propofol dosing regimens for ICU sedation based upon an integrated pharmacokinetic-pharmacodynamic model. Anesthesiology..

[6] Thille AW, Rodriguez P, Cabello B, Lellouche F, Brochard L (2006). Patient-ventilator asynchrony during assisted mechanical ventilation. Intensive Care Med..

[7] Tobin MJ, Jubran A, Laghi F (2001). Patient-ventilator interaction. Am J Respir Crit Care Med..

[8] Goodman NW, Black AM, Careter JA (1987). Some ventilatory effects of propofol as sole anaesthetic agent. Br J Anaesth..

[9] Vaschetto R, Cammarota G, Colombo D, Longhini F, Grossi F, Giovanniello A (2014). Effects of propofol on patient-ventilator synchrony and interaction during pressure support ventilation and neurally adjusted ventilatory assist. Crit Care Med..

[10] Guo TZ, Jiang JY, Buttermann AE, Maze M (1996). Dexmedetomidine injection into the locus ceruleus produces antinociception. Anesthesiology..

[11] Virtanen R, Savola JM, Saano V, Nyman L (1988). Characterization of the selectivity, specificity and potency of medetomidine as an α_2_-adrenoceptor agonist. Eur J Pharmacol..

[12] Hall JE, Uhrich TD, Barney JA, Arain SR, Ebert TJ (2000). Sedative, amnestic, and analgesic properties of small-dose dexmedetomidine infusions. Anesth Analg..

[13] Venn RM, Grounds RM (2001). Comparison between dexmedetomidine and propofol for sedation in the intensive care unit: patient and clinician perceptions. Br J Anaesth..

[14] Venn RM, Hell J, Grounds RM (2000). Respiratory effects of dexmedetomidine in the surgical patient requiring intensive care. Crit Care..

[15] Bloor BC, Ward DS, Belleville JP, Maze M (1992). Effects of intravenous dexmedetomidine in humans. II. Hemodynamic changes. Anesthesiology.

[16] Jakob SM, Ruokonen E, Grounds RM, Sarapohja T, Garratt C, Pocock SJ (2012). Dexmedetomidine versus midazolam or propofol for sedation during prolonged mechanical ventilation: two randomized controlled trials. JAMA..

[17] Boles JM, Bion J, Connors A, Herridge M, Marsh B, Melot C (2007). Weaning from mechanical ventilation. Eur Respir J..

[18] Sinderby C, Navalesi P, Beck J, Skrobik Y, Comtois N, Friberg S (1999). Neural control of mechanical ventilation in respiratory failure. Nat Med..

[19] Venn RM, Bradshaw CJ, Spencer R, Brealey D, Caudwell E, Naughton C (1999). Preliminary UK experience of dexmedetomidine, a novel agent for postoperative sedation in the intensive care unit. Anaesthesia..

[20] Huupponen E, Maksimow A, Lapinlampi P, Sarkela M, Saastamoinen A, Snapir A (2008). Electroencephalogram spindle activity during dexmedetomidine sedation and physiological sleep. Acta Anaesthesiol Scand..

[21] Roukonen E, Parviainen I, Jakob SM, Nunes S, Kaukonen M, Shepherd ST (2009). Dexmedetomidine versus propofol/midazolam for long term sedation during mechanical ventilation. Intensive Care Med..

[22] Schmidt M, Demoule A, Polito A, Porchet R, Aboab J, Siami S (2011). Dyspnea in mechanically ventilated critically ill patients. Crit Care Med..

[23] Gilstrap D, MacIntyre N (2013). Patient–ventilator interactions: implications for clinical management. Am J Respir Crit Care Med..

[24] Murias G, Villagra A, Blanch L (2013). Patient–ventilator dyssynchrony during assisted invasive mechanical ventilation. Minerva Anestesiol..

[25] Vitacca M, Bianchi L, Zanotti E, Vianello A, Barbano L, Porta R (2004). Assessment of physiologic variables and subjective comfort under different levels of pressure support ventilation. Chest..

[26] Schmidt M, Banzett RB, Raux M, Morélot-Panzini C, Dangers L, Similowski T (2014). Unrecognized suffering in the ICU: addressing dyspnea in mechanically ventilated patients. Intensive Care Med..

[27] Hansen-Flaschen JH, Brazinsky S, Basile C, Lanken PN (1991). Use of sedating drugs and neuromuscular blocking agents in patients requiring mechanical ventilation for respiratory failure: a national survey. JAMA..

[28] de Wit M, Pedram S, Best AM, Epstein SK (2009). Observational study of patient–ventilator asynchrony and relationship to sedation level. J Crit Care..

[29] Shehabi Y, Chan L, Kadiman S, Alias A, Ismail WN, Tan MA (2013). Sedation depth and long-term mortality in mechanically ventilated critically ill adults: a prospective longitudinal multicentre cohort study. Intensive Care Med..

[30] Slutsky AS (2010). Neuromuscular blocking agents in ARDS. N Engl J Med..

[31] Vassilakopoulos T, Petrof BJ (2004). Ventilator-induced diaphragmatic dysfunction. Am J Respir Crit Care Med..

[32] Blanch L, Villagra A, Sales B, Montanya J, Lucangelo U, Luján M (2015). Asynchronies during mechanical ventilation are associated with mortality. Intensive Care Med..

